# Hypoglycemia and Dandy-Walker variant in a Kabuki syndrome patient: a case report

**DOI:** 10.1186/s12881-020-01117-8

**Published:** 2020-10-02

**Authors:** Wei Guo, Yanguo Zhao, Shuwei Li, Jingqun Wang, Xiang Liu

**Affiliations:** Department of Neonatology, Xingtai People’s Hospital, Xingtai, 054031 Hebei China

**Keywords:** Kabuki syndrome, Dandy-Walker malformation, Neonatal hypoglycemia, Case report

## Abstract

**Background:**

Kabuki syndrome (KS) is a rare congenital condition with cardinal manifestations of typical facial features, developmental delays, skeletal anomalies, abnormal dermatoglyphic presentations, and mild to moderate intellectual disability. Pathogenic variants in two epigenetic modifier genes, *KMT2D* and *KDM6A,* are responsible for KS1 and KS2, respectively.

**Case presentation:**

A Chinese girl had persistent neonatal hypoglycemia and Dandy-Walker variant. Whole-exome sequencing identified a novel single nucleotide deletion in *KMT2D* (NM_003482.3 c.12165del p.(Glu4056Serfs*10)) that caused frameshift and premature termination. The mutation was de novo. According to the American College of Medical Genetics and Genomics (ACMG) guidelines, this variant is considered pathogenic. The patient was diagnosed with KS by molecular testing.

**Conclusion:**

A single novel mutation in *KMT2D* was identified in a KS patients with hypoglycemia and Dandy-Walker variant in the neonatal stage. A molecular test was conducted to diagnose KS at an early stage.

## Background

Kabuki syndrome (KS, MIM: 147920, 300,867) is a rare condition with an incidence of approximately 1 in 32,000 people [1]. The syndrome is characterized by the following typical facial features: long palpebral fissures with eversion of the lateral third of the lower eyelid, arched and broad eyebrows with the lateral third displaying notching or sparseness, short columella with a depressed nasal tip and large prominent or cupped ears. In addition to distinct facial features, cardinal manifestations also include developmental delays, persistent fetal fingertip pads, infantile hypotonia, mild to moderate intellectual disability and global developmental delay. Other clinical findings have reported congenital heart defects, genitourinary anomalies, cleft lip and/or palate, gastrointestinal anomalies including anal atresia, and ocular and dental anomalies [2, 3]. Pathogenic variants in *KMT2D* and *KDM6A* are responsible for KS 1 (MIM: 147920) [4] and KS2 (MIM: 300867) [5], respectively. *KMT2D* is located on chromosome 12. Pathogenic variants of *KMT2D* are found in approximately 75% of KS cases [6–8], and the inheritance pattern is autosomal dominant. *KDM6A* is located on chromosome X and is inherited as X-linked dominant. Pathogenic variants are found in only 3–5% of KS patients [9–12].

KS is seldom diagnosed during the newborn period. First, the generally consistent features of KS, such as the typical facial features and persistent fetal finger pads, become increasingly obvious as age increases. Second, the typical facial features are less pronounced at the neonatal stage, resulting in many cases going unnoticed by doctors who are not familiar with KS. Here, we report the case of a young girl who experienced hypoglycemia and had Dandy-Walker variant; she was diagnosed with KS1 in the second month after birth by whole-exome sequencing. Dandy-Walker variant has rarely been reported in KS patients before.

## Case presentation

**Fig. 1:**
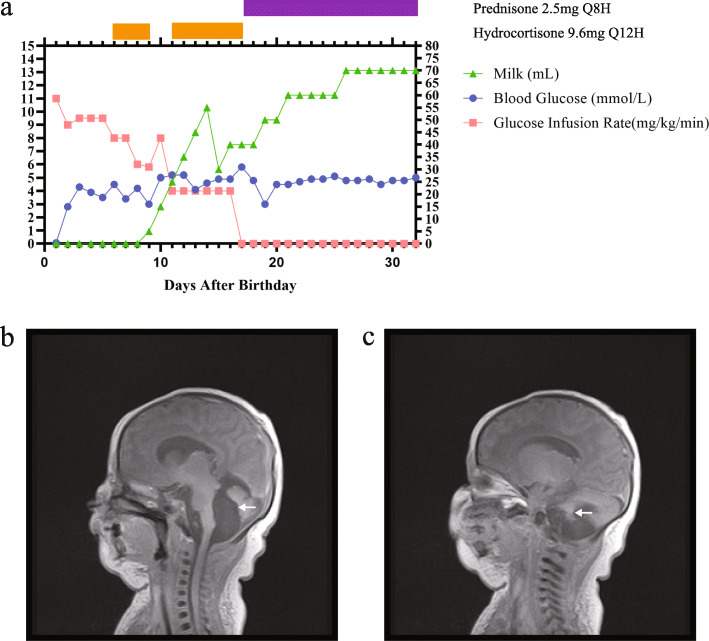
The clinical features of the patient. **a** Blood glucose (mmol/L, left Y axis), glucose infusion rate (mg/kg/min, left Y axis) milk (mL, right Y axis), and medical treatment during the first month in the NICU. **b** Magnetic resonance image of cystic enlargement of the fourth ventricle (arrow). **c** Magnetic resonance image of hypoplasia of the cerebellar vermis (arrow)

**Fig. 2:**
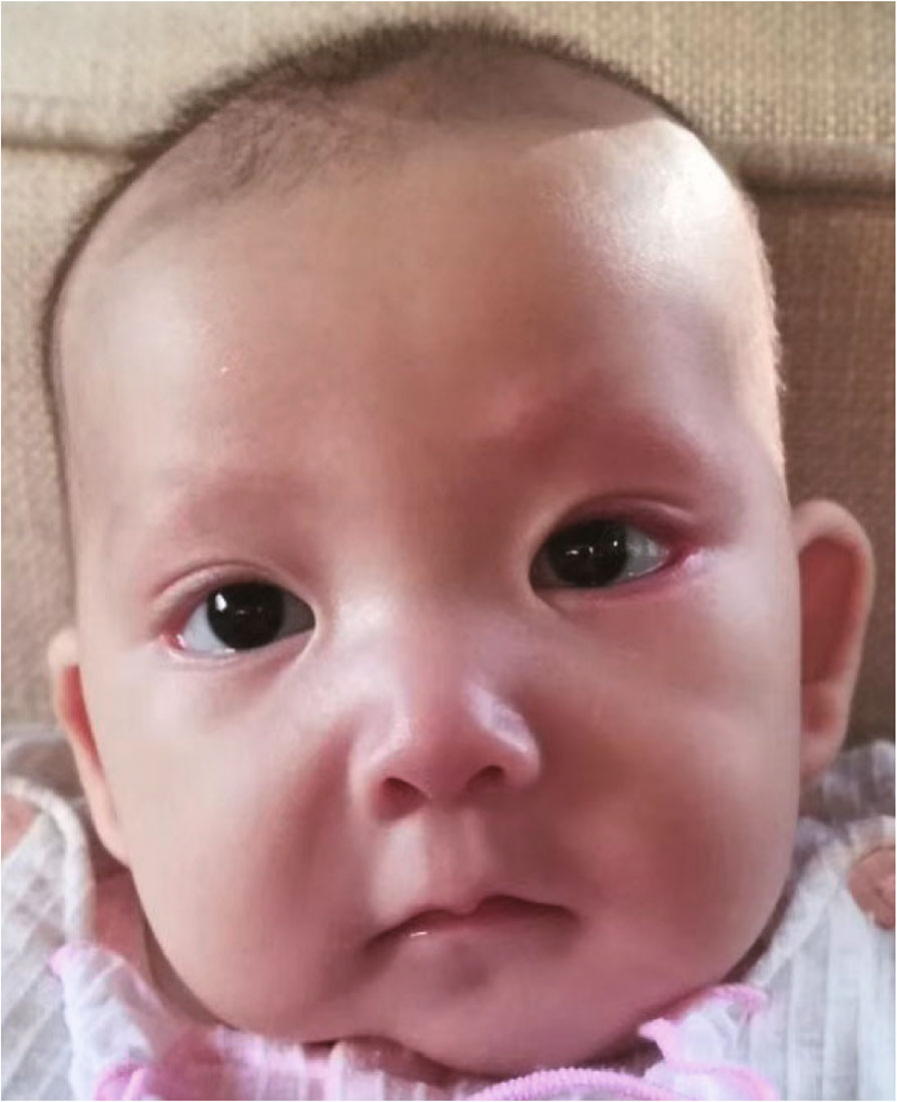
The facial features of the patient at three months old

A young girl was the second child of a nonconsanguineous couple of Chinese ancestry. Her elder sister was normal, and her family history was unremarkable. The patient was naturally conceived when her mother was 36 years old. Nuchal translucency (NT) was 1.1 mm at the first-trimester screening. No apparent defect was observed on a routine second-trimester ultrasound. However, ultrasound examination revealed polyhydramnios at 30 and 37 weeks. The patient was born at 38 weeks gestation by cesarean delivery and had normal birth parameters (Supplementary Table 1). The child’s Apgar scores were 9, 10, and 10 at 0, 5, and 10 min after birth, respectively. Twenty minutes after birth, the patient exhibited cyanosis, hypotonia, decreased spontaneous movements, weak crying and severe hypoglycemia (blood glucose: 0.06 mmol/L) and was therefore transferred to the neonatal intensive care unit (NICU). The blood gas (Supplementary Table 2) and X-ray examinations supported the diagnosis of neonatal respiratory distress syndrome, and she was placed on mechanical ventilation. Treated with a surfactant, she was weaned from mechanical ventilation 7 days later. The liver function test results were normal (Supplementary Table 3). No sign of metabolic acidosis was noticed. However, for 32 days in the NICU, she had persistent hypoglycemia. Constant glucose infusion was needed to maintain her blood glucose in a safe range. Hydrocortisone or prednisone was used to normalize blood glucose (Fig. 1a). Serum insulin and c-peptide testing was performed on the morning of the 9th day. Her serum insulin was normal, but her c-peptide level was elevated (Table 1). Magnetic resonance imaging (MRI) revealed no pituitary gland defect, but it did reveal cystic enlargement of the fourth ventricle and hypoplasia of cerebellar vermis (Fig. 1b and c). She did not pass the auditory brainstem response test. A cardiac ultrasound revealed ductus arteriosus, patent foramen ovale and mild tricuspid regurgitation. When the patient was 3 months old, we examined her facial features considering the criteria of KS, as we had already obtained the diagnosis though genetic testing. Elongated palpebral fissures with eversion of the lower lateral eyelids and prominent ears were observed (Fig. 2).

**Table 1:** Features of hypoglycemia in the patient on the morning of the 9th day after birth

Features	Result
C-peptide (ng/mL)	4.8 (0.3–3.7)
Insulin (uIU/mL)	9.3 (4.0–23.46)
Blood glucose (mmol/L)	5.7 (2.6–6.8)
Cortisol (ng/mL) (8:00 AM)	279.9 (72.6–322.8)
Adrenocorticotropic hormone (ACTH) (pg/mL)	5.2 (5–48)

**Fig. 3:**
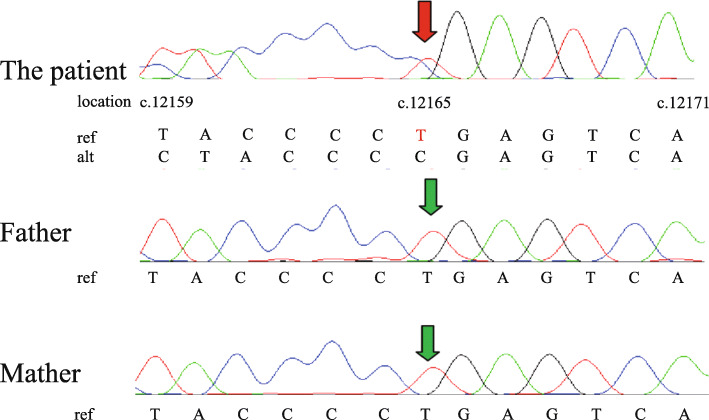
Sanger sequencing results of the patient and her parents. The single nucleotide deletion in the patient is indicated by the red arrow. The same position in her parents is indicated by the green arrow. The reverse primer was used for sequencing, and the mutation was a reverse-complement reading. The “c.” indicates the position in the transcript (NM_003482.3). The red letter “T” in the ref. line of the patient indicates the deletion of a nucleotide

The patient had persistent hypoglycemia, poor feeding, and Dandy-Walker variant, leading to the suspicion of a congenital genetic disorder. After written consent to participate was obtained from the parent. Whole-exome sequencing was performed. Peripheral blood samples were collected from the patient and her parents, and genomic DNA was extracted. The library was constructed by SureSelectXT plus Human All Exon V6 kits (Agilent, Santa Clara, California, USA) and sequenced on a MiSeq platform (Illumina, San Diego, California, USA). The variants with an allele frequency (AF) > 1% in gnomAD felt out. The mutations that were previously reported, considered damaging (nonsense, frameshift, or splicing site) or absent in gnomAD were given priority. The gene set on the basis of Human Phenotype Ontology (HPO) (HP: 0001943) was applied to narrow down the candidate gene list. By analyzing the sequencing data, we found a heterozygous single nucleotide deletion (c.12165del) in the 39th (of 54) exon of *KMT2D* (NM_003482.3), which was predicted to cause coding sequence frameshift and premature translation termination (p.(Glu4056Serfs*10)). The variant was confirmed by Sanger sequencing and was tested for familial segregation demonstrating de novo occurrences (Fig. 3, Supplementary Method). None of the pathogenic variants were found in genes regulating insulin secretion (Supplementary Table 4). The patient was diagnosed with KS1 based on molecular evidence.

## Discussion ﻿and conclusions

KS is a congenital condition characterized by typical facial features, infantile hypotonia, mild to moderate intellectual disability, persistent fetal fingertip pads, and global developmental delay. The diagnosis is traditionally initially made according to the clinical features and then confirmed by molecular diagnosis. However, not all patients have typical clinical features, especially Chinese patients [13]. Therefore, patients often suffer from misdiagnosis or a missed diagnosis. The development of next-generation sequencing has made rapid genetic testing possible and is helpful in the identification and differential diagnoses of rare diseases. We performed whole-exome sequencing on the 9th day after birth and made the diagnosis in the second month after birth. During genetic counseling, we provided information on KS and suggested a relevant examination to the child’s caregivers to rule out undiscovered abnormalities associated with the syndrome. Although KS cannot be cured, early diagnosis improves future health and helps patients reach their full potential though early intervention.

Hypoglycemia is not a cardinal manifestation of KS. Only 8–10% of patients with KS have hypoglycemia during the neonatal or infantile stage [14]. The causes of hypoglycemia in KS patients are attributed to combined pituitary hormone deficiency [15], growth hormone deficiency [16], adrenal insufficiency [17], and congenital hyperinsulinism [16, 18]. In our patient, hypoglycemia was present on the first day of birth and was sustained for almost a month after birth. The glucose infusion rate was initially 11 mg/kg/min and then 9.5 mg/kg/min until hydrocortisone was applied. No pituitary defects were observed. The ACTH and cortisol levels were normal. We observed elevated c-peptide levels. However, hydrocortisone had already been administered, and glucose infusion had been performed when serum c-peptide was tested. Therefore, we could not make a diagnosis of hyperinsulinism-hypoglycemia based on elevated c-peptide levels, but hyperinsulinism was initially suspected.

It has been suggested that KS is correlated with brain anomalies. Various structural brain anomalies, including temporoparietal subarachnoid cyst [19], perisylvian cortical dysplasia [20], unilateral perisylvian cortical dysplasia [21], Arnold Chiari I malformation [22, 23], cerebellar vermis dysplasia [24], corpus callosum dysplasia [22, 25], thinning of the pituitary [13], and polymicrogyria [26, 27], are infrequently reported in KS patients. Among the major structural brain anomalies in KS patients, Dandy-Walker malformations have been reported in three patients [22, 28, 29]. In addition to major anomalies, based on MRI, Jennifer et al. found that the volume of gray matter in the bilateral hippocampus and dentate gyrus in patients with KS was significantly decreased compared those in healthy controls [30]. MRI of our patient showed cystic enlargement of the fourth ventricle and hypoplasia of the cerebellar vermis, which fulfilled the diagnostic criteria of Dandy-Walker variant. Similar to KS, Dandy-Walker malformation is also a rare condition, with an incidence of approximately 2.74 per 100,000 live births [31]. Dandy-Walker malformation has already been reported in three KS patients. Dandy-Walker variant was not be independent of KS in our patient. The mechanism of KS is still unclear. Additional cases and analyses are needed to evaluate the causes of brain anomalies in KS patients.

The *KMT2D* mutation spectrum includes nonsense mutations, frameshift mutations, missense mutations, splicing site mutations, gross deletions, gross duplications, and indels, among which more than 60% are nonsense mutations and frameshift mutations [21]. Therefore, the loss of function is the mechanism of disease development. Our patient had the c.12165del variant, which also led to frameshift and premature termination. Familial segregation demonstrated a de novo mutation. This variant was not found in gnomAD, mbiobank [32], ClinVar or the Human Gene Mutation Database (HGMD) (2019.4) or included in the literature on the comprehensive pathogenic mutation spectrum [33]. According to the ACMG guidelines, the variant is classified as pathogenic considering PVS1, PM2, and PM6 [34].

In conclusion, this case study reports an early diagnosis of KS1 in a patient with the atypical phenotype, hypoglycemia and Dandy-Walker variant by genetic testing. These data broaden the mutation spectrum and add information about phenotypes relating to KS. A molecular diagnosis should be considered for patients suspected of having genetic disorders to discover and differentiate rare diseases.

## Supplementary information

**Additional file 1: Supplementary Table 1.** Birth parameter. **Supplementary Table 2.** Blood gas result. **Supplementary Table 3.** Liver function test. **Supplementary Table 4.** The variants in genes regulating insulin secretion of coding region and splicing region(±10).

**Additional file 2.** Supplementary Method.

## Data Availability

All data supporting the results reported in a published article can be found. The raw datasets generated and/or analysed during the current study are not publicly available in order to protect participant confidentiality; however, are available from the corresponding author on reasonable request. The reference sequence for validation of the c.12165del in the *KMT2D* gene was acquired from the NCBI Nucleotide database (https://www.ncbi.nlm.nih.gov/nucleotide/) by using accession number NM_003482.3. And the reference sequence for validation of variants in ABCC8, KCNJ11, GLUD1, GCK, HADH, SLC16A1, HNF1A in supplementary Table 4 were acquired from the NCBI Nucleotide database() by using accession number NM_000352.5, NM_000525.3, NM_005271.5, NM_000162.5, NM_005327.6, NM_003051.3, NM_000545.6 respectively. The allele frequency was acquired from the website of correspond website: gnomAD (https://gnomad.broadinstitute.org/), mbiobank (http://www.mbiobank.com/). The pathogenicity of variants was searched in clinvar (https://www.ncbi.nlm.nih.gov/clinvar/), and Human Gene Mutation Database (http://www.hgmd.cf.ac.uk/). The gene set was acquired from the website of The Human Phenotype Ontology (https://hpo.jax.org/) by using the accession number HP:0001943.

## Abbreviations

KS: Kabuki syndrome
NICU: Neonatal intensive care unit
ACMG: The American College of Medical Genetics and Genomics
HPO: The Human Phenotype Ontology
HGMD: Human Gene Mutation Database
