# Supplementary material for: Hypoglycemia and Dandy-Walker variant in a Kabuki syndrome patient: a case report
Source: BMC Med Genet. 2020 Oct 2;21:193. doi: 10.1186/s12881-020-01117-8 (PMC7531129; doi:10.1186/s12881-020-01117-8)
Supplement: Supplementary file 1 — Additional file 1: Supplementary Table 1. Birth parameter. Supplementary Table 2. Blood gas result. Supplementary Table 3. Liver function test. Supplementary Table 4. The variants in genes regulating insulin secretion of coding region and splicing region(±10). [file 12881_2020_1117_MOESM1_ESM.docx]

**Supplementary Material**

Supplementary Table1 Birth parameter

| Features | Result |
| --- | --- |
| Weight(g) | 3840 |
| Length(cm) | 51 |
| Head circumference(cm) | 35 |
| Chest circumference(cm) | 33 |
| Temperature(℃) | 36.5 |
| Blood Pressure(mmHg) | 77/31 |

Supplementary Table2 Blood gas result

| Features | Result |
| --- | --- |
| PH | 7.23(7.35-7.45) |
| PaCO_2_(mmHg) | 66.9(35-45) |
| PaO_2_(mmHg) | 33(50-70) |
| HCO_3_^-^(mmol/L) | 18.8(21-25) |
| ABE(mmol/L) | -5.7(-3-3) |
| Lactate(mmol/L) | 1.75(1.22-2.66) |

Supplementary Table3 Liver function test

| Feature | Result |
| --- | --- |
| ALT(U/L) | 7.2 (7-40) |
| AST(U/L) | 64.6 (13-35) |
| GGT(U/L) | 70.6(7-45) |
| Alkaline phosphatase(U/L) | 150.8(40-160) |
| Total bilirubin(μmol/l) | 71.2(1.0-23.0) |
| Direct bilirubin(μmol/l) | 9.4(0.1-8.6) |
| Total Protein(g/L) | 57.3(65-86) |
| Albumin(g/L) | 39.6(40-55) |

Supplementary Table 4 The variants in genes regulating insulin secretion of coding region and splicing region(±10)

| Gene | Location | Type | HGVS | AF (gnomAD) | SIFT | Revel | Mutation Taster | PROVEAN |
| --- | --- | --- | --- | --- | --- | --- | --- | --- |
| ABCC8 | chr11:17418477 | homo | NM_000352.5:c.4105G>T (p.Ala1369Ser) | 0.6434 | Tolerated | Benign | Polymorphism | Neutral |
| ABCC8 | chr11:17419279 | het | NM_000352.5:c.3819G>A (p.Arg1273=) | 0.2995 | - | - | - | - |
| ABCC8 | chr11:17436868 | het | NM_000352.5:c.2274G>A (p.Ala758=) | 0.00001823 | - | - | - | - |
| ABCC8 | chr11:17449929 | homo | NM_000352.5:c.1947G>A (p.Lys649=) | 0.1724 | - | - | - | - |
| ABCC8 | chr11:17452492 | het | NM_000352.5:c.1686C>T (p.His562=) | 0.4231 | - | - | - | - |
| ABCC8 | chr11:17496516 | het | NM_000352.5:c.207T>C(p.Pro69=) | 0.4719 | - | - | - | - |
| KCNJ11 | chr11:17409572 | homo | NM_000525.3:c.67A>G (p.Lys23Glu) | 0.6477 | Tolerated | Benign | Polymorphism | Neutral |
| KCNJ11 | chr11:17409069 | het | NM_000525.3:c.570C>T (p.Ala190=) | 0.2572 | - | - | - | - |
| KCNJ11 | chr11:17408630 | homo | NM_000525.3:c.1009G>A (p.Val337Ile) | 0.6453 | Tolerated | Benign | Polymorphism | Neutral |
| GLUD1 | chr10:88820789 | homo | NM_005271.5:c.942A>G (p.Leu314=) | 0.1223 | - | - | - | - |
| GCK | chr7:44185088 | het | NM_000162.5:c.1253+8C>T(p.?) | 0.279 | - | - | - | - |
| HADH | chr4:108931039 | homo | NM_005327.6:c.257T>C (p.Leu86Pro) | 0.9152 | Tolerated | Benign | Polymorphism | Neutral |
| SLC16A1 | chr1:113456546 | homo | NM_003051.3:c.1470T>A (p.Asp490Glu) | 0.5996 | Tolerated | Benign | Polymorphism | Neutral |
| HNF1A | chr12:121437382 | homo | NM_000545.6:c.1720A>G (p.Ser574Gly) | 0.9954 | Tolerated | Benign | Polymorphism | Neutral |
