# Supplementary material for: Hypoglycemia and Dandy-Walker variant in a Kabuki syndrome patient: a case report
Source: BMC Med Genet. 2020 Oct 2;21:193. doi: 10.1186/s12881-020-01117-8 (PMC7531129; doi:10.1186/s12881-020-01117-8)
Supplement: Supplementary file 2 — Additional file 2. Supplementary Method. [file 12881_2020_1117_MOESM2_ESM.docx]

**Supplementary** **Method**

Method for sanger sequencing

A fragment of 517bp containing mutation was amplified by Extaq using primer:

Forward: 5’-TAGGCATGATTCCAACCCCA-3’

Reverse: 5’-CTGACGGGCAAGGAACAAAA-3’.

The reaction was assembled following manufacturer's instruction.

The PCR program

| Temperature | Time | Cycle |
| --- | --- | --- |
| 94℃ | 5min | 1 |
| 94℃ | 30s | 10 |
| 65-55℃(touch down 1℃ per cycle) | 30s |  |
| 72℃ | 30s |  |
| 94℃ | 30s | 30 |
| 55℃ | 30s |  |
| 72℃ | 30s |  |
| 72℃ | 5min | 1 |
| 12℃ | hold |  |

The fragment was sequenced using ABI 3500. The reverse primer was used for sequencing.
